# ‘*We are short-staffed*’: Dialysis practitioners’ infection prevention and control adherence challenges in the City of Tshwane, South Africa

**DOI:** 10.4102/sajid.v41i1.796

**Published:** 2026-04-08

**Authors:** Siyanda A. Ngema, Thabiso A. Bale, Livhuani M. Nezingahe, Tendani S. Ramukumba

**Affiliations:** 1 Adelaide Tambo School of Nursing Science, Faculty of Science, Tshwane University of Technology, Tshwane, South Africa

**Keywords:** HD practitioners, haemodialysis, infections, infection prevention and control, challenges, adherence

## Abstract

**Background:**

The burden of infections in dialysis facilities continues to rise worldwide. Due to the invasive procedures associated with haemodialysis (HD), patients undergoing HD in dialysis facilities are at high risk of acquiring bloodstream-related infections.

**Objective:**

The present study explored the practices of dialysis practitioners regarding the prevention of HD bloodstream-related infections at selected dialysis facilities in the City of Tshwane, Gauteng province, in South Africa.

**Methods:**

An exploratory-descriptive contextual qualitative study was conducted in five dialysis facilities utilising semi-structured interviews. Purposive sampling was conducted until data saturation was reached from 10 in-depth semi-structured interviews conducted with dialysis practitioners.

**Results:**

Data analysis was conducted concurrently with data collection using a qualitative thematic analysis approach, whereby Tesch’s eight-step method was used to analyse the data. One theme, infection prevention challenges of dialysis practitioners, emerged with three subthemes: (1) in-service training, (2) shortage of resources and (3) colleagues’ behaviors and practices.

**Conclusion:**

A lack of staffing, training and resources hinders the implementation of infection prevention and control (IPC) to ensure optimal care in dialysis facilities. These findings suggest that enhancing training programmes and promoting a culture of compliance with IPC protocols are crucial for improving patient safety and outcomes in dialysis settings.

**Contribution:**

This study highlights contextual insights into the infection prevention challenges faced by dialysis practitioners in South African dialysis facilities, highlighting critical gaps in training, resources and staff compliance that inform targeted interventions to reduce bloodstream infections.

## Introduction

Haemodialysis (HD) is the predominant modality for kidney replacement therapy (KRT) globally,^1,2^ particularly prevalent in both the public and private healthcare sectors, where its utilisation rates stand at 41% and 84%, respectively.^1^ Notably, HD accounts for 87% of all chronic dialysis patients, underscoring its critical role in managing kidney failure (KF).^1^ However, the invasive nature of HD predisposes patients to a heightened risk of acquiring hospital-acquired infections (HAIs).^3,4^ Amongst these infections, access-related bloodstream infections (BSIs) represent a significant complication. In the National Healthcare Safety Network report from 2014 in the United States, the access-related BSI rate was 0.49 per 100 patient-months, which varied by access type: 0.16 for fistulas, 0.27 for grafts and 1.83 for central venous catheters (tunnelled and non-tunnelled).^3^

The global burden of infections in dialysis facilities is escalating, marking infections as leading causes of morbidity and mortality in HD populations.^4^ A third of patients undergoing chronic dialysis develop infections, and many have been hospitalised within 90 days.^5,6,7^ A concerning trend observed globally indicates that, for every 100 patients in acute-care hospitals, seven in high-income countries and 15 in low-income and middle-income countries contract one or more HAIs, with one in ten succumbing to their infection.^6,7^

Amidst these challenges, multiple organisations have recommended approaches and programmes to mitigate risks associated with HAIs and combat antimicrobial resistance.^6,7,8,9,10^ The Sustainable Development Goals (SDGs), especially Goal 3, which targets good health and well-being by 2030, emphasise the prevention of infectious diseases as essential for effective healthcare. The goals also emphasise the urgent need to address global health issues, such as antimicrobial resistance.^10^ Considering these urgent priorities, South Africa has developed a strategic framework to enhance patient safety, minimise HAIs and improve overall health outcomes, asserting the necessity for equitable access to superior health services for all citizens.^8^

The high burden of HAI in sub-Saharan Africa (SSA) varies between regions.^11^ Yet, there is a lack of detailed data on HAIs, emphasising the need for infection prevention and surveillance approaches. Similar to other African countries, South Africa lacks comprehensive statistics on infections within dialysis facilities, highlighting a significant gap in understanding the challenges faced by healthcare workers in Africa. The 2022 South African Renal Registry (SARR) annual report documented 9342 patients receiving chronic dialysis or kidney transplants nationwide by December 2022, a 5.4% increase from 8866 patients in 2021.^12^ These data indicate a persistent upward trend in KRT demand, highlighting a growing public health challenge within the South African healthcare system.^12^

Given the complex dynamics between patients and healthcare providers in dialysis facilities, where multidisciplinary collaboration is essential, it is vital to explore the challenges healthcare workers encounter in adhering to IPC measures. There is a paucity of studies focusing on the challenges faced by dialysis healthcare workers in complying with and adhering to IPC measures in the City of Tshwane. This study explored practices of preventing catheter-related infections amongst dialysis practitioners at five dialysis facilities in the City of Tshwane.

## Research methods and design

### Design and context

A qualitative, exploratory-descriptive and contextual research design^13^ was used to explore the practices of dialysis nurses and clinical technologists, referred to in this study as HD practitioners, regarding the prevention of infections associated with dialysis vascular catheters in the HD units. The study comprised five HD units, including four private and one public HD unit. All the selected HD units serve diverse HD populations within the City of Tshwane.

### Population and sampling process

Dialysis nurses and clinical technologists were chosen for this study due to their critical roles in managing patients undergoing haemodialysis. These professionals possess first-hand experience and expertise in managing dialysis vascular catheters, which are essential components of the treatment process. Their involvement directly impacts patient safety and outcomes, particularly concerning the prevention of infections associated with these catheters. By focusing on this specific group, the study aimed to gather in-depth insights into their practices, challenges and strategies regarding infection prevention and control (IPC).

Using a purposeful sampling method, 10 HD practitioners were selected, recruited and interviewed using semi-structured interviews. This segment of the study represents the second phase of the overarching research project. Participants were selected based on their voluntary consent to engage in the study. Additionally, the researchers verified each participant’s years of experience in the dialysis field to ensure a comprehensive representation. By purposefully including both highly experienced and relatively less experienced HD practitioners, a nuanced array of insights and perspectives was obtained from the participants.^14^

### Recruitment

Participants were recruited after their involvement in the quantitative phase of the study, which included a survey of HD practitioners across five HD units in the City of Tshwane. Individuals who expressed interest in participating further were invited based on their willingness and professional experience. The aim was to include a diverse sample of HD practitioners, from those new to the field to those with extensive experience. The recruitment process emphasised voluntary participation, providing candidates with clear information about the study’s objectives and procedures. Ten HD practitioners were selected to provide rich qualitative data on IPC practices related to dialysis vascular catheters.

This helped facilitate the collection of rich qualitative data regarding the practitioners’ experiences, challenges and perspectives on HD practices.^14^ Interviews were conducted in English, using both remote online platforms and face-to-face meetings, whilst adhering to the ethical considerations of informed consent and confidentiality. The semi-structured interview guide centred on the principal question: ‘*What are your experiences in the haemodialysis facility you work in regarding IPC practices related to dialysis vascular catheter infections?*’ Probing questions were employed to further elicit detailed responses, whereby the interviews lasted between 13 min and 45 min each.

### Data analysis

The researchers itemised audiotapes and read transcripts to gain a deeper understanding of the recorded interviews and further familiarise themselves with the collected data. All data were transcribed verbatim. Data analysis involved multiple steps, including reading notes, identifying emergent themes and clustering insights into subthemes. Two researchers independently coded the transcripts using descriptive thematic analysis following Tesch’s methodology.^15^ Manual coding continued until data saturation was reached,^14^ with relevant quotes selected to illustrate the findings. Validity was reinforced through independent assessments of the themes and subthemes by the researchers.

### Ethical considerations

Approval was obtained from the Tshwane University of Technology’s Research Ethics Committee (REC Ref#: 2020/02/004), with gatekeeper permissions secured from all participating HD facilities. Participants were fully informed about the study’s purpose and procedures before the commencement of data collection. Informed consent was obtained, ensuring confidentiality and anonymity using pseudonyms. The study specifically targeted HD practitioners who actively connect and disconnect patients from HD machines and other dialysis-related activities. Data collection occurred across five HD facilities to capture a wide range of experiences.

## Results

Data were collected from participants working across all five selected HD units. Participants had voluntarily agreed to participate in the semi-structured interviews from the online survey by providing their email addresses. Semi-structured interviews were conducted between June 2021 and September 2021, spanning 4 months. Table 1 depicts the demographics of participants.

**TABLE 1:** Demographics of participants.

Pseudo name	Years working in nephrology	Highest qualification	Professional status
P1	19	Postgraduate Diploma in Nephrology Nursing	Nurse
P2	13	Postgraduate Diploma in Nephrology Nursing	Nurse
P3	5	Postgraduate Diploma in Nephrology Nursing	Nurse
P4	4	Diploma in Nursing	Nurse
P5	9	Postgraduate Diploma in Nursing	Nurse
P6	23	BTech Clinical Technology	Clinical technologist
P7	5	Diploma in Clinical Technology	Clinical technologist
P8	35	Postgraduate Diploma in Nephrology Nursing	Nurse
P9	21	Postgraduate Diploma in Nephrology Nursing	Nurse
P10	8	BTech in Clinical Technology	Clinical technologist

P, participant.

The participants were predominantly nurses, with qualifications primarily in Nephrology Nursing, including several with postgraduate diplomas. The ages of the participants ranged from 28 years to 57 years, indicating a diverse range of perspectives and maturity in the field. Notably, the years of experience in nephrology varied significantly from 4 years to a substantial 35 years, suggesting a range of practical knowledge and expertise amongst the group. In terms of qualifications, the majority had pursued advanced education in nursing, reflecting a commitment to specialised training in nephrology. Additionally, clinical technologists were represented in this cohort, with backgrounds in clinical technology, enhancing the interdisciplinary nature of nephrology care across HD units. This composition of professionals underscores the importance of both nursing and clinical technology in managing kidney-related health issues and highlights the varied yet complementary roles these experts play within the healthcare system. Such diversity in age, experience and educational background was deemed to contribute to a well-rounded approach to patient care in nephrology.

### Thematic presentation of results

This section explores a central theme of the study: the challenges faced by HD practitioners. Accompanying this theme are three significant subthemes that provide deeper insight, namely a lack of in-service training, the nuances of staff behaviour and practices in various situations and the implications of staffing shortages. These elements provide a comprehensive picture of the respondents’ challenges in HD unit environments. Table 2 delineates the breakdown of the theme, subthemes and categories and uses illustrative quotes to describe each subtheme. The participants were referred to using the letter ‘P’ with the corresponding number as pseudonyms.

**TABLE 2:** Theme, subthemes and categories.

Theme	Subtheme	Categories
1. Infection prevention challenges for dialysis practitioners	1.1. In-service training	Lack of in-service training Infrequent training opportunities
	1.2. Shortage of resources	Shortage of personnel Shortage of supplies for IPC
	1.3. Colleagues’ behaviours and practices	Impact on infection control practices Effects of situational factors

IPC, infection prevention and control.

#### Subtheme 1.1: In-service training

Participants were asked about the in-service training they received regarding the prevention of dialysis catheter-related infections, infection prevention in HD facilities and strategies that could be effectively implemented to mitigate infection risks. Many cited a significant lack of training and resources as critical factors contributing to deficiencies in practice. Whilst participants acknowledged having undergone some training related to IPC at various points in their roles, there was unanimous agreement on the pressing need for more comprehensive and frequent training sessions in HD facilities. Insights from the participants reflected their experiences as quoted below:

A participant expressed a concern:

‘We do not have in-service training anymore, when we did, it was at least once a month, but I can’t even recall the last time I received such training.’ (P6)

This sentiment was echoed by a participant who reminisced about their initial onboarding, saying:

‘I only got training when I came for orientation; after that, there has been nothing.’ (P8)

In contrast, a participant, who has been working in acute dialysis for just 2 months, lamented:

‘I have not yet received any training related to infection prevention this year. Last year, at the chronic facility, we also received no training in this vital area.’ (P1)

Participants collectively underscored the importance of in-service training as essential for promoting IPC, advocating that it could significantly improve adherence to protocols in dialysis facilities.

A participant said:

‘The training at my workplace is scheduled, but this year I didn’t attend. I don’t remember the last time I last had one [*training*], but the last in-service training that I attended was about donning and doffing of PPE, and I do find them [*in-service training*] relevant.’ (P5)

The perceived inadequacy of training was also a critical factor influencing staff practices. *P5* noted that though training sessions were scheduled, attendance had been inconsistent. This lapse in training opportunities suggests a lack of knowledge refreshment, which may contribute to complacency in the following established procedures. *P2* reinforced this notion by suggesting that comfort over time leads to lax adherence to protocols.

Furthermore, participants reported varied experiences regarding the frequency of training, revealing inconsistent schedules and attendance of training related to IPC in their work environment. A glaring disparity emerged amongst HD practitioners regarding the intervals of such training. The comments provided below revealed this gap.

Some participants said:

‘I only got the training when I came for orientation, then after that, no.’ (P8)

‘The last training I attended, I think, was six months ago; no, it’s been eight months now.’ (P4)

‘People forget … maybe they do not realise the significance of infection control. They often focus solely on connecting and disconnecting patients without following the necessary steps.’ (P9)

‘Training is not conducted frequently at work because we are considered competent. I would say ongoing training occurs every three [*months*] to four months. The last in-service training I participated in was about five months ago, and it primarily concentrated on COVID-19 precautions, specifically how to prevent cross-infections between patients.’ (P6)

These insights underscore the critical need for systematic and regular training in IPC to ensure that all practitioners in HD facilities are knowledgeable and consistently follow best practices.

#### Subtheme 1.2: Shortages of resources

Participants noted that a shortage of human resources and supplies is increasingly compromising their ability to adhere to infection prevention precautions for dialysis vascular catheters, ultimately affecting the quality of patient care. As highlighted by participants, these shortages create an environment where healthcare workers often find themselves overworked and unable to effectively perform their duties.

A participant reflected on the frustrations expressed by many when they noted that such conditions can prevent compliance with IPC measures:

‘Sometimes you find that we are working, and we are short-staffed, so on those days we are doing the wrong way, most of the time.’ (P10)

Another participant added that:

‘I think it is because of the shortage of staff, like when we are short-staffed, let me say we have 12 patients, then you’re three staff members.’ (P4)

Moreover, P3 emphasised that inadequate staffing and limited resources not only hinder the proper execution of infection prevention procedures, but also contribute to a cycle of stress and inefficiency amongst personnel. This persistent issue, echoed by various participants, calls for urgent attention and action to ensure that healthcare professionals are adequately supported in their roles.

One participant highlighted that:

‘We are mostly short-staffed because you don’t get enough chance or enough time to do the whole procedure properly, sometimes there are not enough supplies, so you think people are not doing the right thing, but sometimes it’s because of shortage of staff or shortage of supplies.’ (P3)

Participants expressed that they envision a situation where they combat non-compliance, which may be better if given enough staff and supplies.

A participant alluded that:

‘They must just provide us with enough staff ’cause sometimes we become overworked, and you know when you become overworked you struggle to do your job to the fullest ability, so staffing is a challenge as well as supplies.’ (P3)

Regarding the shortage of supplies a participant said:

‘Sometimes you find people running around with aprons from this patient to another patient in the ward and, people will tell you that there is shortage, but at some point, now with this COVID thing, we are really short of PPEs, am sure it is shortage, shortage of staff has been a problem for years and years.’ (P1)

#### Subtheme 1.3: Colleagues’ behaviour and practices

This subtheme reveals significant insights into the behaviours and practices of HD practitioners regarding the prevention of HD catheter-related infections. Multiple key practices and staff behaviours that emerged from the participants’ responses include inadequate hand hygiene, a tendency to take shortcuts and the impact of situational factors on compliance with infection control protocols.

Several participants expressed concerns about their colleagues’ practices, specifically regarding hand hygiene.

For instance, a participant said:

‘Sometimes it becomes a bit difficult because we are mostly short-staffed ’cause you don’t get enough chance or enough time to do the whole procedure properly. Sometimes in between patients we don’t wash our hands.’ (P3)

In addition, a participant stated that the challenge of non-compliance is common with all members of the multidisciplinary team and cited that:

‘There are times when nurses do not follow the steps as set out in the policy. Then in that regard then there are gaps that needs to be filled in that regard. And the doctors, only few would spray or wash their hands in between patients during their rounds.’ (P2)

A participant recalled and remarked on her experience of a lack of thorough hand-washing amongst staff, indicating a potential risk for infection transmission.

Another participant further said that:

‘I have worked with someone who doesn’t wash hands accordingly, so because we’ve got this screening thing, connecting a catheter needs an aseptic technique, you need to wash your hands, very thoroughly, so they just pulled the screen on, go wash hands like your social hand washing or something go behind the screen, they’ll be touching all over.’ (P1)

Similarly, a participant expressed doubts about their colleagues’ commitment to implementing appropriate IPC measures, highlighting a collective sense of discomfort about the standards maintained within the team:

‘I think people tend to get comfortable, and then they do not follow procedure all the time. That is why it is good to always have in-service training to remind one another about the importance of following the correct procedure to avoid those kind of things where people tend to fall into the trap of being too relaxed.’ (P2)

The participants’ narratives indicated that situational factors significantly impact adherence to guidelines. Whilst busy periods detract from strict compliance, calmer times allow staff to follow procedures diligently. A recurring sentiment amongst the participants was the inclination to take shortcuts due to workload, particularly in high-pressure situations.

A participant openly shared an experience of prioritising speed over compliance due to a busy environment, confessing that:

‘I don’t wanna lie, we tend to take the shortcut because we busy, we want to cover many patients as we want, so some they will not even put the sterile gloves, but it depends with the situation, when it is a calm situation and we not busy, we go through the procedure because we try to avoid infection … [*Y*]es and the cross infection.’ (P4)

This statement underscores how work pressures related to extreme workloads can compromise infection control practices.

Participants also remarked on how the fast-paced dialysis environment can sometimes lead them to bypass the infection prevention measures due to wanting to meet the demands. In this regard,

A participant highlighted that:

‘We have a tendency again of not wearing a mask when attending to the patient, or wearing goggles when attending to the patient, and sometimes when we feel like it’s a minor challenge, it is also easy for us to attend to it without gloves. Like swapping your lines maybe from your arterial to the venous or from the venous to arterial. We sometimes do that without wearing gloves. That is also very common in dialysis.’ (P6)

## Discussion

This study explored IPC practices and challenges amongst dialysis practitioners at five dialysis facilities in the City of Tshwane. Staff behaviours and practices regarding the prevention of HD catheter-related infections illustrate a complex interplay between individual diligence, situational pressures and training inadequacies. It is crucial to prevent harm to patients, healthcare professionals and visitors due to infections in healthcare settings to achieve quality care, patient safety and healthcare security, and to decrease HAIs and antimicrobial resistance.^16^

### Shortage of resources

Shortages of staff and a lack of supplies emerged as contributing factors to non-compliance with optimal IPC practices in the HD facilities. These findings of barriers concur with other findings in developing countries, where suboptimal provision of supplies in dialysis settings has been reported.^17^ These breaches in practice may occur when HD practitioners experience a shortage of staff in the facilities whilst having to meet the demands of patients and end up lacking in compliance with guidelines for the prevention of dialysis vascular catheter-related infections. HD facilities should always be equipped with optimal staff-to-patient ratios and equipment to facilitate implementation and adherence to evidence-based guidelines.

Despite the importance of staffing in HD settings, staffing and its importance in optimal patient care has been noticeably overlooked. In the current study, HD practitioners reported that dialysis facilities are short-staffed, which affected their adherence to IPC measures, as in a study from Northern Nigeria.^18^ Single-patient-centred care and adherence to the nurse-to-patient ratio must be implemented and enforced to limit the risk of transmission.^19^ Globally, there is an increase in staffing challenges within the nephrology sector especially in low-income countries, where a shortage of all types of HD practitioners, including nephrologists, surgeons, radiologists and nurses, is prevalent.^20^

### Training

It is unclear whether there was a lack of IPC training in HD facilities in the current study or whether practitioners did not attend training when it was organised. A descriptive cross-sectional study conducted in HD centres in Sudan revealed low levels of training attendance in training courses about IPC in HD.^21^ A study in Palestine found inadequate adherence to hand hygiene, PPE and infection control practices, with participants lacking proper training in IPC protocols.^22^ A study in Saudi Arabia found that despite the availability of personal protective equipment (PPE), there was a significant lack of PPE usage and poor adherence to recommended practices by nurses in HD facilities.^23^Training may enhance adherence to IPC by training staff in dialysis facilities to ensure compliance with IPC measures.^16,24^ A study in Egypt revealed a significant increase in the practice scores of precautionary measures, including handwashing, glove usage and wearing face masks, amongst nurses in Egypt after the educational interventional programme compared to before the implementation of the programme.^23^

### Behavioural patterns

In this study, multiple key practices and staff behaviours emerged from participants’ responses, including inadequate hand hygiene, tendencies to take shortcuts and the impact of situational factors on compliance with IPC protocols. This is supported by a Vietnam study,^19^ which asserted that the breakdown in providing nursing care activities on a patient-centred basis was a response to the limited supply of gloves and hand hygiene facilities, in addition to pressures from patients on nurses to provide dialysis services without delay.^19^ A quantitative longitudinal study in South Africa, which used covert direct observation, revealed lower compliance with hand hygiene amongst nurses and clinical technologists providing HD services.^25^ Other observational studies have shown that nurses tend to ignore hand hygiene and are not consistently committed to wearing clean gloves as required.^19,26^ The results indicate that situational factors have a significant impact on adherence to guidelines. However, some IPC guidelines developed internationally may not apply to less developed HD facilities, necessitating the need for locally developed guidelines.

Haemodialysis practitioners face multiple challenges that hinder effective, optimal IPC practices and compliance. Corresponding with the current study’s findings are studies from Vietnam and South Africa, which highlight significant challenges that HD practitioners face in adhering to hygiene protocols.^19,25^ Inadequate hand hygiene^19^ illustrates how limited supplies, such as gloves and hand hygiene facilities, negatively impact patient-centred care. Compounding this issue are patients’ demands, which can lead to shortcuts in adhering to hygiene practices. The South African observations revealed low compliance with the WHO’s five moments for hand hygiene,^25^ emphasising the need for better training and resources.

This study’s findings cannot be generalised because they are contextual. Only five HD facilities were involved; therefore, the reality of other HD facilities might differ. The study was also conducted at the peak of the coronavirus disease 2019 (COVID-19) pandemic. Another notable limitation of this study is the absence of detailed operational data regarding the participating HD units. Information on key facility characteristics, including public versus private-sector distribution, hospital-attached versus standalone configurations, unit occupancy rates, nurse-to-patient staffing ratios and vascular access profiles (proportion of patients dialysing via arteriovenous fistulae versus central venous catheters), was not systematically collected. These contextual factors may significantly influence infection rates and HD staff practices, potentially limiting the generalisability and transferability of our findings to other HD settings. Whilst the South African Nephrology Society (formerly the South African Renal Society) recommends a staff ratio of one nurse or clinical technologist per four patients for dialysis facilities, in the current study, staff ratios were not measured.^27^ Future multicentre studies should incorporate comprehensive facility-level data collection to enable more nuanced interpretation of infection surveillance results and facilitate meaningful benchmarking across diverse HD environments.

Whilst the current study focused on nurses and dialysis technologists as frontline personnel with direct catheter care responsibilities, the exclusion of nephrologists represents a notable limitation. Though nephrologists have less frequent patient contact, they critically influence infection risk through key decisions on vascular access, antimicrobial prescribing and catheter management once infection occurs. These clinical decisions directly shape the situational pressures and contextual factors examined in this study. Future research would benefit from incorporating nephrologists’ perspectives to capture the complete decision-making pathway influencing dialysis catheter-related bloodstream infection rates in HD settings.

To enhance IPC compliance in dialysis facilities, several key recommendations can be implemented. First and foremost, comprehensive in-service training programmes focused on IPC protocols should be established for all dialysis practitioners. This will ensure that ongoing education and awareness of best practices are prioritised, allowing staff to stay informed about the latest guidelines. Furthermore, addressing staffing shortages is critical. Facilities can hire additional personnel or optimise task delegation to reduce the workload on existing staff, which will enable them to concentrate more effectively on IPC measures. Alongside staffing changes, it is essential to provide practitioners with adequate resources, including sanitation supplies and equipment. This support will facilitate compliance with IPC guidelines. Establishing a culture of safety within dialysis units is equally crucial. In such an environment, adherence to protocols should be prioritised and recognised, reinforcing the importance of IPC compliance amongst all staff members. Finally, conducting further research is necessary to identify specific barriers to effective IPC practices. This research will help develop tailored interventions that meet the unique needs of practitioners, particularly in regions such as the City of Tshwane.

## Conclusion

In conclusion, this is the first study to explore the challenges dialysis practitioners face in IPC in the City of Tshwane. Significant gaps, such as a lack of staffing, training and resources in the HD units, hinder the implementation of IPC measures to ensure optimal care in dialysis facilities. These challenges reveal a pressing need for better staffing, formal and informal training and resources to promote positive adherence and compliance and prevent dialysis-related vascular catheter infections. These implementations will improve patient safety and outcomes in dialysis settings. Addressing these deficiencies requires targeted interventions and heightened awareness amongst healthcare providers. Additionally, further investigation into the impact of staffing on infection rates is essential to inform clinical practice guidelines and policies regarding optimal dialysis staffing.
